# Associations between dietary patterns and the risk of breast cancer: a systematic review and meta-analysis of observational studies

**DOI:** 10.1186/s13058-019-1096-1

**Published:** 2019-01-29

**Authors:** Yunjun Xiao, Junjie Xia, Liping Li, Yuebin Ke, Jinquan Cheng, Yaojie Xie, Winnie Chu, Polly Cheung, Jean Hee Kim, Graham A. Colditz, Rulla M. Tamimi, Xuefen Su

**Affiliations:** 1 Department of Molecular Epidemiology, Shenzhen Center for Disease Control and Prevention, Shenzhen, Guangdong China; 2 MPH Education Center, Shantou University Medical College, Shantou, Guangdong China; 3 School of Nursing, The Hong Kong Polytechnic University, Hong Kong, China; 4 Department of Imaging & Interventional Radiology, Faculty of Medicine, The Chinese University of Hong Kong, Hong Kong, China; 5 Hong Kong Breast Cancer Foundation, Hong Kong, China; 6 School of Public Health and Primary Care, Faculty of Medicine, The Chinese University of Hong Kong, Hong Kong, China; 7 Alvin J. Siteman Cancer Center and Department of Surgery, Washington University School of Medicine, St. Louis, MO USA; 8 Channing Division of Network Medicine, Brigham and Women’s Hospital, Harvard Medical School, Boston, MA USA; 9 Department of Epidemiology, Harvard T.H. Chan School of Public Health, Boston, MA USA

**Keywords:** Dietary patterns, Breast cancer, Observational studies, Meta-analysis

## Abstract

**Background:**

Epidemiologic evidence suggests that certain dietary patterns were associated with breast cancer risk, but the results have been inconclusive. We assessed the associations between different dietary patterns and the risk of breast cancer by conducting a meta-analysis of observational studies.

**Methods:**

Relevant articles were searched in PubMed, Embase, and Cochrane library databases through September 2017. Multivariable-adjusted relative risks (RRs) and 95% confidence intervals (CIs) comparing the highest and lowest categories of Western and prudent dietary patterns were combined by using the random-effects meta-analyses.

**Results:**

We identified 32 eligible articles including 14 cohort and 18 case-control studies (34 Western and 35 prudent studies). The pooled analyses found that a Western dietary pattern was associated with a 14% increased risk (RR 1.14, 95% CI 1.02, 1.28), whereas a prudent dietary pattern was associated with an 18% reduced risk of breast cancer (RR 0.82, 95% CI 0.75, 0.89). In addition, sub-group analyses showed that the positive association between a Western dietary pattern and breast cancer risk was significant among postmenopausal (RR 1.20, 95% CI 1.06, 1.35), but not premenopausal women (RR 1.18, 95% CI 0.99, 1.40), and significant for hormone receptor-positive tumors (RR 1.18, 95% CI 1.04, 1.33), but not receptor-negative tumors (RR 0.97, 95% CI 0.83, 1.12). In contrast, the inverse association between a prudent dietary pattern and breast cancer was significant in premenopausal (RR 0.77, 95% CI 0.61, 0.98), but not postmenopausal women (RR 0.88, 95% CI 0.74, 1.03), and significant for both hormone receptor-positive and receptor-negative tumors.

**Conclusions:**

The results of the current meta-analysis suggest a possible increased risk of breast cancer associated with a Western dietary pattern and a reduced risk with a prudent dietary pattern. Large-scale cohort studies with a high quality need to be conducted to further confirm the findings of the current meta-analysis. As dietary patterns are modifiable, these findings may provide viable strategies for breast cancer prevention through changes in dietary intake.

**Electronic supplementary material:**

The online version of this article (10.1186/s13058-019-1096-1) contains supplementary material, which is available to authorized users.

## Background

Breast cancer is the most commonly diagnosed cancer and the leading cause of cancer death among women in both developed and developing countries. Globally, the incidence rate of breast cancer has been rising rapidly over the past few decades [1]. Most of the well-established breast cancer risk factors, such as family history, age at menarche, age at menopause, and reproductive history, e.g., age at first birth and parity, are, in general, not readily modifiable [2]. Migrant studies suggest that potentially modifiable lifestyle factors, in particular diet, also play an important role in breast cancer prevention [3].

A substantial number of epidemiological studies have examined the associations between individual foods and the risk of breast cancer. High intakes of red meat, animal fats, and refined carbohydrates have been shown to be associated with an increased risk [4–6], whereas intake of fruits, vegetables, whole grains, and dietary fiber has been linked with a reduced risk of breast cancer [6–8]. However, foods contain many nutrients and the different nutrients interact with each other. Although these individual food items have been associated with breast cancer in some instances, the totality of evidence is inclusive, as supported by the World Cancer Research Fund (WCRF) Report on Nutrition and Physical Activity [9]. Therefore, dietary patterns, which are derived from factor analysis and/or principal component analysis, have been adopted and considered as better indicators of overall dietary intake and nutritional status than individual food items.

Numerous epidemiological studies have assessed the associations between different dietary patterns and the risk of breast cancer. Some studies have found a positive association between a Western dietary pattern and breast cancer risk [10, 11], and others observed an inverse association between prudent or healthy dietary patterns and breast cancer risk [12, 13]. However, contradictory results have also been reported [11, 14]. In addition, no association between different dietary patterns and the risk of breast cancer has been reported in some other studies [15–17]. A previous meta-analysis published in 2010 which included 8 case-control studies and 8 cohort studies showed that a prudent/healthy dietary pattern was associated with a reduction in the risk of breast cancer, but no association between a Western/unhealthy dietary pattern and breast cancer risk. Since then, additional 16 original observational studies have been published including 6 cohort and 10 case-control studies, more than double the number of breast cancer cases (*n* = 43,285 vs. 19,908 cases) than in the previous meta-analysis. Considering the inconsistent results in the current evidence and the insufficient statistical power of the previous meta-analysis due to the small number of studies and breast cancer cases, we conducted an updated meta-analysis of observational studies to review and summarize the epidemiologic evidence on the association between dietary patterns and the risk of breast cancer. We further examined these associations by study designs and characteristics of study populations.

## Methods

### Search strategy

We followed the PRISMA standard guidelines to perform the meta-analyses of observation studies and write the manuscript according to the PRISMA checklist (Additional file 1). PubMed, EMBASE, and Cochrane Library databases were searched through September 2017 for relevant articles that reported the association between different dietary patterns and the risk of breast cancer. To avoid missing any relevant study, reference lists and review papers on this topic were also reviewed. The following keywords or phrases were used in the structured literature search, including “diet”, or “dietary”, or “pattern”, or “risk” in combination with “breast”, or “breast cancer”, or “breast neoplasm”, or “cancer”, or “tumor”, or “carcinoma”, or “neoplasm”, or “mammary”, and “human” to search articles published in English.

### Study selection

Studies were included if they met the following inclusion criteria: (1) it was a case-control or a cohort study; (2) the exposure of interest was the most common dietary consumption pattern derived from factor analysis and/or principal component analysis. To reduce the heterogeneity across the studies, only the dietary patterns with similar factor loadings of foods were selected. For example, the Western or Western-like dietary pattern with high loadings of foods including red and/or processed meats, high-fat dairy products, potatoes, and sweets was selected as a representative unhealthy dietary pattern, whereas a prudent or similar dietary pattern with high loadings of foods such as fruits, vegetables, fish, whole grains, and low-fat dairy products was considered as a healthy dietary pattern; (3) the outcome of interest was incident breast cancer cases excluding recurrent cases; all incident breast cancer cases were diagnosed and verified by pathological biopsies or other standard methods, with controls/non-cases being females without breast cancer; all breast cancer types were included such as in situ or invasive cancer; (4) the relative risks (RRs), hazards ratios (HRs), or odds ratios (ORs) and the corresponding 95% CI for the highest compared with the lowest category of dietary patterns were reported.

Two reviewers independently screened the titles and abstracts of the searched papers and excluded the articles which did not meet the above-described inclusion criteria. For those that were difficult to determine their eligibility, a full-text assessment was conducted. All disputes, if any, were resolved by discussion.

**Fig. 1:**
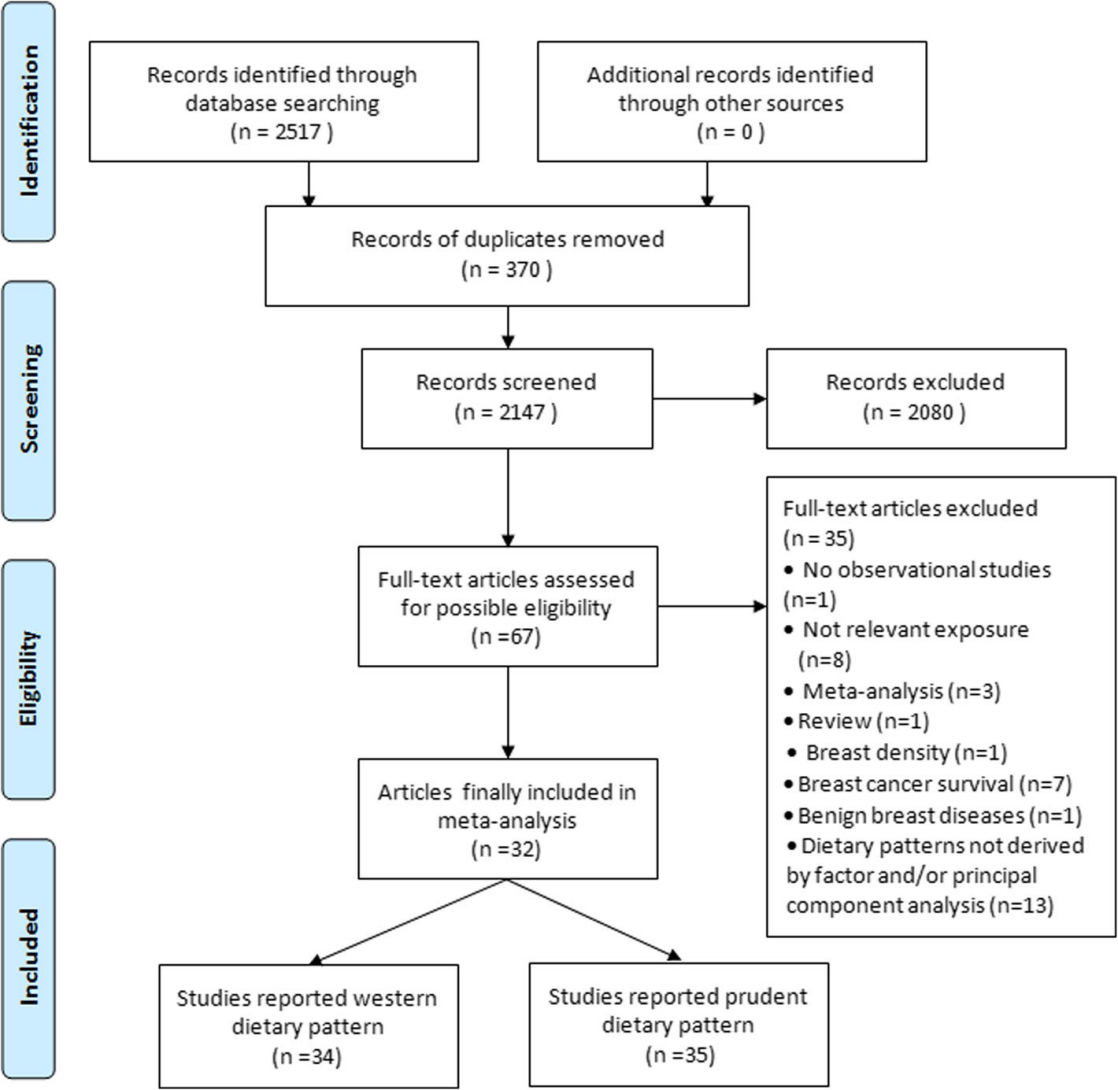
Flow chart of literature search and selection of studies on dietary patterns and the risk of breast cancer

We initially identified 2517 potentially relevant articles from the databases, and 370 records were excluded because they were duplicates. After title and abstract review, 2080 articles were further excluded. After reviewing the full text of the remaining 67 articles, 35 papers were excluded due to the following reasons: one article was not an observational study [18]; eight studies did not assess the relevant exposure of dietary patterns [19–26]; three were meta-analyses [8, 27, 28]; one was a review paper [29]; one study reported breast density as the outcome [30]; one used benign breast disease as the outcome [31], seven studies looked at breast cancer survival, not breast cancer risk [32–38], and an additional 13 papers did not use dietary patterns that were derived by factor analysis and/or principal components analysis [39–51]. Because one article reported on two cohort studies [52] and another article reported on three cohort studies within the single article [14], finally, 32 eligible articles that reported 34 studies (17 case-control and 17 cohort studies) of Western and 35 studies (18 case-control and 17 cohort studies) of prudent dietary patterns with breast cancer risk were included in the current meta-analysis. A flow chart of the study selection process is presented in Fig. 1.

### Data extraction and quality assessment

Two reviewers independently abstracted data on study characteristics and results by using a standardized data collection form. Discrepancies in data extraction between the reviewers were resolved by consensus. Data extracted included the following: first author’s last name; year of publication; location of the study; study design; sample size; average age of participants; dietary assessment methods; dietary patterns; RRs, HRs, and ORs with the corresponding 95% CIs from the fully adjusted model for the highest compared with the lowest category of dietary patterns; and potential confounders adjusted in the multivariate analysis.

We also systematically assessed the study quality. Briefly, a nine-score system on the basis of the Newcastle Ottawa Scale (NOS) was used to assess the quality of the included studies. Each study was evaluated on three broad criteria: (1) the proper selection of study population, (2) the comparability of the study groups, and (3) the ascertainment of the exposure or outcome of interest. Two reviewers independently assessed the quality of each study. Disagreements were resolved through discussion to reach a consensus. Studies scored greater or equal to 7, out of a maximum 9 points, were considered to be high-quality studies.

### Statistical analyses

RRs were used as a common measure of the association between dietary patterns and the risk of breast cancer across studies. HRs, ORs, or incidence rate ratios (IRRs) were directly considered as estimates of RR. To calculate summary RR and its 95% CI, we pooled the results by using random-effects meta-analysis. The random-effects analysis was chosen a priori because of the anticipated clinical and methodological heterogeneity and because it is considered more conservative than the fixed-effects analysis, as it accounts for both within- and between-study heterogeneity [53]. Heterogeneity across studies was evaluated by using the Q statistics at the *P* < 0.10 level of significance. We also calculated the *I*^2^ statistic, which describes the total variation across studies attributable to heterogeneity rather than chance; an *I*^2^ value greater than 50% indicates at least moderate heterogeneity [54].

Because participant characteristics and confounder adjustment differed across the studies, which may result in different associations between dietary patterns and breast cancer risk among the studies, we further conducted stratified analyses to explore possible sources of heterogeneity and to examine the influence of various inclusion criteria on the overall risk estimate. Pre-specified subgroup analyses by menopausal status, hormone receptor status, study design, number of breast cancer cases, number of adjusted variables, quality scores of studies, and with or without adjustment for certain risk factors were performed to assess whether these variables modify the overall risk estimate. We also conducted a sensitivity analysis to investigate the influence of each individual study or a group of studies, for example, studies that used diet history questionnaires, or with the low quality score, on the overall risk estimate by removing one study or a group of studies at a time.

Potential publication bias was assessed by visual inspection of Begg’s funnel plots in which the log RRs were plotted against their standard errors (SEs). We also performed Begg’s rank correlation test and Egger’s linear regression test [55, 56]. All analyses were performed using STATA version 11.0 (StataCorp LP, College Station, Texas). A *P* value < 0.05 was considered statistically significant, except where otherwise specified.

## Results

### Study characteristics

The characteristics of the included 32 articles are presented in Table 1. Eighteen articles reported results from case-control studies [11, 12, 15, 16, 57–70], and 14 articles reported findings from cohort studies [10, 13, 14, 17, 52, 71–79], of which one article reported on two cohort studies [52] and another article reported on three cohort studies within the single article [14]. Therefore, there are a total of 18 case-control studies and 17 cohort studies included in the current meta-analysis. The articles were published between 2001 and 2016. Of these, 11 studies were conducted in North America, 4 in South America, 11 in Europe, and 8 in Asia. Sample sizes of studies ranged from 274 to 91,779. The number of breast cancer cases varied from 100 to 4140. Dietary intake was assessed using food-frequency questionnaire (FFQ) in 33 studies and diet history questionnaire in two studies [10, 11]. A wide range of potential confounding factors were adjusted, including age at interview, age at menarche, age at first delivery, body mass index (BMI), smoking, alcohol consumption, energy intake, family history of breast cancer, physical activity, hormone use, and menopausal status.

**Table 1:** Characteristics of the studies included in the systematic review and meta-analysis of dietary patterns and breast cancer risk

Study	Location	Age of participants^1^	Study design	Sample size (number of menopausal status)	Diet assessment method	Dietary patterns identified	Confounding factors adjusted for in the multivariable analysis
Mourouti et al., 2015 [70]	Greece	56 ± 12	Case-control	250 cases (84 premenopausal women/166 postmenopausal women)/250 controls (91 premenopausal women/158 postmenopausal women)	FFQ (86 questions)	Unhealthy food pattern, healthy/prudent pattern, and olive oil and fish pattern	Years of education, family history of breast cancer, BMI, IPAQ score, smoking ever, and menopausal status, and place of living
Castello et al., 2014 [67]	Spain	No reported	Case-control	973 cases (513 premenopausal women /460 postmenopausal women)/973 controls (551 premenopausal women/422 postmenopausal women)	FFQ (117 questions, past 5 years)	Western pattern, prudent pattern, Mediterranean pattern, Alternate Healthy Index, and Alternate Mediterranean Diet Score	Total calories, alcohol consumption, BMI, average physical activity in the past year, smoking, education, previous history of breast disease other than cancer, family history of breast cancer, age at menarche, age at first delivery, and menopausal status
Tumas et al., 2014 [69]	Argentina	58 ± 12	Case-control	100 cases (22 premenopausal women/78 postmenopausal women) /294 controls (78 premenopausal women/216 postmenopausal women)	FFQ (127 questions, past 5 years)	Traditional, rural, prudent, and starchy	BMI, educational level, total energy intake, gynecological status, and physical activity
Karimi et al., 2013	Iran	30–65	Case-control	100 cases (63 premenopausal women/37 postmenopausal women) /174 controls (109 premenopausal women/65 postmenopausal women)	FFQ (168 questions, past 1-year)	Healthy dietary pattern, and unhealthy dietary pattern	Age and menopausal status, age at menarche, age at first full-term pregnancy, smoking status, oral contraceptive drug use, BMI, physical activity, family history of breast cancer, and relative accuracy of energy reporting
Bessaoud et al., 2012 [15]	France	28–85	Case-control	437 cases (no reported) /922 controls (no reported)	FFQ (162 questions)	Mediterranean, Western, meat-eaters, and drinkers	Total energy intake, education, parity, breast-feeding age at first full-term pregnancy, duration of ovulatory activity, BMI, physical activity, and first-degree family history of breast cancer
Demetriou et al., 2012 [66]	Greece	40–70	Case-control	935 cases/817 controls (all are postmenopausal women)	FFQ (32 questions)	Fruit/vegetables/fish	Age at interview, family history, age at first full term pregnancy, HRT use, exercise, age at menarche, height, BMI, and PCA derived patterns 1, 2 and 3 in post-menopausal women only
Buck et al., 2011 [64]	Germany	50–74	Case-control	2884 cases/5509 controls (all are postmenopausal women)	FFQ (176 questions, past 1-year)	Healthy pattern and unhealthy pattern	Year of birth, study center, menopausal induction, BMI, education level, first-degree family history of breast cancer, history of benign breast disease, number of pregnancies, age at menarche, breastfeeding history, total number of mammograms, smoking habit, and total energy
Zhang et al., 2011 [65]	China	25–70	Case-control	438 cases (306 premenopausal women/132 postmenopausal women)/438 controls (295 premenopausal women/143 postmenopausal women)	FFQ (81 questions, past 1-year)	Vegetable-fruit-soy-milk-poultry-fish, and refined grain-meat-pickle	Age at menarche, live births and age at first live birth, months of breast feeding, BMI, history of benign breast disease, mother/sister/daughter with breast cancer, physical activity, passive smoking, and total energy intake
Ronco et al., 2010 [20, 63]	Uruguay	40–75	Case-control	111 cases (11 premenopausal women/100 postmenopausal women) /222 controls (24 premenopausal women/198 postmenopausal women)	FFQ (120 questions, past 5-year)	Low-fat, fried white meat, non-alcoholic beverages, Western, fatty cheese, and prudent	Age, education, physical activity, family history of breast cancer among first-degree relatives, BMI, smoking, drinking, age at menarche, parity, menopausal status, total energy intake, and scored patterns each for the others
Cho et al., 2011	Korea	25–77	Case-control	357 cases (216 premenopausal women/141 postmenopausal women)/357 controls (228 premenopausal women/129 postmenopausal women)	FFQ (103 questions, past 1-year)	Vegetables-seafood and meat starch	Age, BMI, family history of breast cancer, current use of dietary supplements, education, occupation, smoking, alcohol intake, physical activity, menopausal status (if applicable), age at menarche, parity, total energy intake, and postmenopausal hormone use for postmenopausal women
Wu et al., 2009 [12]	USA	25–74	Case-control	1248 cases (no reported) /1148controls (no reported)	FFQ (174 questions, usual intake)	Western meat/starch; Ethnic meat/starch; and vegetable-soy	Age, Asian ethnicity, education, birthplace, years of residence in the USA, years of physical activity, marital status, parity, age at menarche, type of menopause, age at menopause, and recent BMI
De Stefani et al., 2009 [61]	Uruguay		Case-control	461 cases (no reported)/2532 controls (no reported)	FFQ (64 questions, usual intake)	Prudent, drinker, traditional, and western	Age, residence, urban/rural status, education, BMI, smoking status, years since stopping smoking, number of cigarettes/d among current smokers, total energy intake, and main food groups for the individual dietary patterns
Murtaugh et al., 2008 [11]	USA	25–79	Case-control	757 cases (315 premenopausal women/442 postmenopausal women) /867 controls (312 premenopausal women/555 postmenopausal women) (Hispanic); 1524 cases (538 premenopausal women/986 postmenopausal women)/1598 controls (492 premenopausal women/1106 postmenopausal women) (non-Hispanic)	Diet-history Questionnaire (computerized–interviewer administered)	Western, prudent, Native Mexican, Mediterranean, and dieter	Age, center, education, family history of breast cancer, smoking, total activity, calories, dietary fiber, dietary calcium, height, parity, recent hormone exposure, BMI, interaction of recent hormone exposure and BMI
Edefonti et al., 2008 [60]	Italy	17–79	Case-control	2569 cases (987 premenopausal women/1579 postmenopausal women)/3413 controls(1074 premenopausal women/2334 postmenopausal women)	FFQ (78; 2 years before diagnosis/hospital admission (controls)	Animal products, vitamins and fiber, unsaturated fats, and starch-rich	Age, education, parity, menopausal status, geographic area, BMI, history of female cancers, history of digestive cancers, and energy intake
Hirose et al., 2007 [59]	Japan	40–79	Case-control	1885 cases (no reported)/22,333controls (10,577 premenopausal women/11756 postmenopausal women)	FFQ (13 diet factors, 17 food items; 1 year before diagnosis/interview (controls))	Prudent, fatty, Japanese, and salty	Age, visit year, motivation, BMI, menopausal status, parity, age at first full-term pregnancy, age at menarche, smoking, drinking, family history of breast cancer, and exercise
Cui et al., 2007 [16]	China	25–64	Case-control	1459 cases (952 premenopausal women/507 postmenopausal women)/1556 controls (990 premenopausal women/566 postmenopausal women)	FFQ (76; past 5 years)	Vegetable-soy and meat-sweet	Age, total energy, family history of breast cancer, history of fibroadenoma, age at menarche, live births, age at first live birth, menopausal status, age at menopause, physical activity during the past 10 y, waist-hip ratio, and education
Ronco et al., 2006 [58]	Uruguay	30–89	Case-control	442 cases (84 premenopausal women /358 postmenopausal women)/442 controls (90 premenopausal women/352 postmenopausal women)	FFQ (64; usual intake)	Traditional, healthy, Western, stew, high-fat, and drinker	Age, residence, urban/rural status, education, family history of breast cancer among first-degree relatives, menopausal status, age at menarche, parity, and total energy intake
Nkondjock and Ghadirian, 2005 [57]	Canada	35–79	Case-control	414 breast cancer cases (no reported)/429 controls (no reported)	FFQ (985; 2 years before diagnosis, corresponding time for controls)	Chocolate–cereal, pork-processed meat, and drinker	Total energy intake, family history of cancer, marital status, physical activity, smoking, BMI, age (at first full-term pregnancy for breast cancer), history of benign breast disease, full-term pregnancies
Shin et al., 2016 [78]	Japan	57 ± 8	Cohort (14·6)	49,552 (718 cases: 185 premenopausal women/533 postmenopausal women)	FFQ (147; previous year)	Prudent, Westernized, and traditional	Age, public healthcare center area, log-transformed energy intake, BMI, smoking status, leisure-time physical activity, total physical activity, age at menarche, parity, age at first birth, menopause status, and use of exogenous female hormones
Harris et al., 2016 [17]	USA	41.0 ± 4.5	Cohort (22)	45, 204 (1477 cases:863 premenopausal women/614 postmenopausal women)	FFQ (124 items; 1960 to 1980, for adolescent diet; 130 items in 1991, 1995, 1999, 2003, and 2007 for adult diet)	Prudent, Western, fast food, AHEI	Age, high-school total calories, height at age 18, age at menarche, BMI at age 18, physical activity in adolescence and family history of breast cancer. Age at first birth/parity, oral contraceptive use, physical activity in adulthood, alcohol consumption, weight change since age 18 and history of benign breast disease, menopausal status/age at menopause, and hormone use.
Kojima et al., 2016	Japan	55.5 ± 9.6	Cohort (16.9)	23,172 (119 cases: 48 premenopausal women/71 postmenopausal women)	FFQ (39 items)	Vegetable pattern, animal food pattern, and dairy product pattern	Age, area, tobacco smoking status, drinking status, family history of breast cancer, age at menarche, age at first birth, parity, energy intake, hormone therapy, daily walking, education, and BMI
Catsburg et al., 2015 [52]	Canada (CSDLH)	49–72	Cohort (13)	39,532 (1496 cases: 591 premenopausal women/625 postmenopausal women)	FFQ (166 items, past 1-year)	Healthy, ethnic, meat and potatoes	BMI, calorie intake, physical activity, family history, and each dietary pattern
	Canada (NBSS)	40–59	Cohort (23)	49,410 (3659 cases: 1795 premenopausal women/1864 postmenopausal women)	FFQ (86-item, usual intake)	Healthy, ethnic, meat and potatoes	BMI, calorie intake, physical activity, family history, and each dietary pattern
Link et al., 2013 [13]	USA	42–60	Cohort (14.1)	91,779 (4140 cases: 1780 premenopausal women/1821 postmenopausal women)	FFQ (103 items, 1-year before baseline)	Plant-based, high-protein, high-fat, high-carbohydrate, ethnic, salad and wine	Race-ethnicity/birthplace, family history of breast cancer, age at menarche, parity/age at first full-term pregnancy, average daily caloric intake, physical activity, socioeconomic status, history of a benign breast biopsy and its interaction with time-dependent age, BMI, height, menopausal status/hormone therapy use, and the other four dietary patterns
Baglietto et al., 2011 [77]	Australia	27–76	Cohort (14.1)	20,967 (815 cases: 285 premenopausal women/530 postmenopausal women)	FFQ (121 items)	Vegetable, fruit and salad, traditional Australian, and meat	Country of birth, age at menarche, parity, duration of lactation, oral contraceptive use, HRT use, menopausal status at baseline, physical activity, alcohol, smoking, level of education, total energy intake, and BMI
Cottet et al., 2009 [10]	France	53 ± 6	Cohort (9.7)	63,374 (2381 cases: all are postmenopausal women)	Diet history questionnaire (208 foods and beverages)	Alcohol/Western and healthy/Mediterranean	Age, education, region at baseline, BMI, height, family history of breast cancer, age at menarche, age at first full-term pregnancy, number of live births, menopausal hormone therapy, history of benign breast disease, lobular carcinoma in situ, oral contraceptive use, breastfeeding history, frequency of Papanicolaou testing, physical activity, smoking status, energy intake excluding alcohol intake, use of phytoestrogen supplement, and use of vitamin /mineral supplements
Agurs-Collins et al., 2009 [76]	USA	38.5 ± 10.6	Cohort (22)	50,778 (1094 cases: 509 premenopausal women/442 postmenopausal women)	FFQ (68 items)	Western and prudent	Age, BMI, alcohol intake, education, age at menarche, parity, age at first birth, family history of breast cancer, strenuous physical activity, energy intake, menopausal status, smoking status, and female hormone use
Velie et al., 2005 [75]	USA	62 ± 8	Cohort (8)	40,559 (1868 cases: no reported)	FFQ (61 items; past year)	Vegetable-fish/poultry-fruit, beef-pork starch, traditional Southern	Age, total energy intake, education, family history of breast cancer, BMI, height, parity, age at first live birth, age at menarche, menopausal hormone use, average weekday vigorous physical activity, smoking status, and alcohol intake
Adebamowo et al., 2005 [73]	USA	26–46	Cohort (8)	90,638 (710 cases: all are premenopausal women)	FFQ [133 items (1991); 142 items (1995); past year]	Prudent and Western	Age at menarche, parity, age at first birth, family history of breast cancer, history of benign breast disease, oral contraceptive use, alcohol intake, energy intake, current BMI, height, smoking habit, physical activity, and multivitamin use
Fung et al., 2005 [74]	USA	30–55	Cohort (16)	71,058 (3026 cases: all are postmenopausal women)	FFQ (116 items; past year)	Prudent and Western	Age, smoking status, BMI, multivitamin use, energy intake, physical activity, family history of breast cancer, history of benign breast disease, duration of and age at menopause, use of hormone replacement therapy, age at menarche, parity, age at first birth, BMI at 18 y of age, weight change since 18y of age, height, and alcohol intake
Mannisto et al., 2005	Netherlands (NLCS)	55–69	Cohort (7)	1598 (1127 cases: no reported)	FFQ (150 items; past year)	Vegetable; pork, processed meat, and potatoes	Age, BMI, height, education, smoking, family history of breast cancer, age at menarche, age at menopause, age at first birth, ever use of oral contraceptive, ever use of hormone replacement therapy, alcohol intake, and energy
	Italy (ORDET)	35–69	Cohort (9)	10,788 (212 cases: no reported)	FFQ (107 items; past year)	Vegetable; pork, processed meat, and potatoes	Age, BMI, height, education, smoking, family history of breast cancer, ever use of oral contraceptive, ever use of hormone replacement therapy, alcohol intake, and energy
	Sweden (SMC)	40–74	Cohort (13)	66,651 (1932 cases: no reported)	FFQ (67 items; past half year)	Vegetable; pork, processed meat, and potatoes	Age, BMI, education, family history of breast cancer, age at first birth, parity, alcohol intake, and energy
Sieri et al., 2004 [72]	Italy	34–70	Cohort (9.5)	8984 (207 cases: no reported)	FFQ (107 items; past year)	Salad vegetable, Western, canteen, and prudent	Age, energy intake, education, parity, height, age at menarche, smoking, and menopausal status
Terry et al., 2001 [71]	Sweden	40–76	Cohort (9.6)	61,463 (1328 cases: no reported)	FFQ (67 items; past half year)	Western, healthy, and drinker	Age, energy intake, BMI, education, family history, parity, and age at first birth

AHEI, Alternative Healthy Eating Index; BMI, body mass index; CSDLH, Canadian Study of Diet, Lifestyle and Health; FFQ, food frequency questionnaire; HRT, hormone replacement therapy; IPAQ, International Physical Activity Questionnaire; NBSS, National Breast Screening Study; NLCS, Netherlands Cohort Study on Diet and Cancer; ORDET, Ormoni e Dieta nella Eziologia dei Tumori; PCA, principal component analysis; SMC, Swedish Mammography Cohort

^1^Values are mean ± SD or age range

Table 2 shows the methodological quality of the included studies. The NOS scores ranged from 6 to 9, with 30 high- and 5 low-quality studies. Bias related to exposure assessment and selection bias were found, and non-response rates were not reported in most case-control studies; bias related to exposure assessment was also found, and follow-up rates were not reported in most cohort studies.

**Table 2:** Assessment of study quality included in the meta-analysis by Newcastle Ottawa Scale (NOS)

Case-control studies	Case definition	Selection of cases	Selection of controls	Definition of controls	Control for most important factor^1^	Control for any additional factor^2^	Ascertainment of exposure	Same method of ascertainment for cases and controls	Non-response rate	Total scores
Mourouti et al., 2015 [70]	1	1	1	1	1	1	0	1	1	8
Castello et al., 2014 [67]	1	1	1	1	1	1	1	1	0	8
Tumas et al., 2014 [69]	1	0	1	1	1	1	1	1	1	8
Karimi et al., 2013	1	0	0	1	1	1	1	1	1	7
Bessaoud et al., 2012 [15]	1	1	1	0	1	1	1	1	0	7
Demetriou et al., 2012 [66]	1	1	1	1	1	1	1	1	0	8
Buck et al., 2011 [64]	1	0	0	0	1	1	1	1	0	5
Zhang et al., 2011 [65]	1	1	0	1	1	1	1	1	1	8
Ronco et al., 2010 [20, 63]	1	0	1	1	1	1	1	1	1	8
Cho et al., 2011	1	1	0	1	1	1	1	1	1	8
Wu et al., 2009 [12]	1	1	1	1	1	1	1	1	1	9
De Stefani et al., 2009 [61]	1	1	0	1	1	1	1	1	1	8
Murtaugh et al., 2008 [11]	1	1	1	1	1	1	1	1	0	8
Edefonti et al., 2008 [60]	1	1	0	1	1	1	1	1	1	8
Hirose et al., 2007 [59]	1	1	0	1	1	1	0	1	0	6
Cui et al., 2007 [16]	1	1	1	0	1	1	1	1	1	8
Ronco et al., 2006 [58]	1	1	0	1	1	1	0	1	1	7
Nkondjock and Ghadirian, 2005 [57]	1	1	1	0	1	1	1	1	1	8
Cohort studies	Selection of exposed cohort	Selection of non-exposed cohort	Ascertainment of exposure	Outcome was not present as baseline	Control for most important factor^1^	Control for any additional factor^2^	Assessment of outcome	Adequate follow-up period for outcome	Adequacy of follow up of cohorts	Total scores
Shin et al., 2016 [78]	1	1	0	1	1	1	1	1	0	7
Harris et al., 2016 [17]	0	1	1	1	1	1	1	1	0	7
Kojima et al., 2016	1	1	0	1	1	1	1	1	0	7
Catsburg (CSDLH), 2015	1	1	0	1	1	1	1	1	0	7
Catsburg (NBSS), 2015	1	1	0	1	1	1	1	1	0	7
Link et al., 2013 [13]	0	1	0	1	1	1	1	1	0	6
Baglietto et al., 2011 [77]	1	1	1	1	1	1	1	1	0	8
Cottet et al., 2009 [10]	1	1	0	1	1	1	1	1	1	8
Agurs-Collins et al., 2009 [76]	1	1	0	1	1	1	1	1	1	8
Velie et al., 2005 [75]	1	1	0	1	1	1	1	1	1	8
Adebamowo et al., 2005 [73]	0	1	0	1	1	1	1	1	1	7
Fung et al., 2005 [74]	0	1	1	1	1	1	1	1	1	8
Mannisto (NLCS), 2005	1	1	0	1	1	1	1	1	0	7
Mannisto (ORDET), 2005	0	1	0	1	1	1	1	1	0	6
Mannisto (SMC), 2005	1	1	0	1	1	1	1	1	0	7
Sieri et al., 2004 [72]	0	1	0	1	1	1	1	1	0	6
Terry et al., 2001 [71]	1	1	0	1	1	1	1	1	1	8

1 denote one score, 0 denote 0 score

CSDLH, Canadian Study of Diet, Lifestyle and Health; NBSS, National Breast Screening Study; NLCS, Netherlands Cohort Study on Diet and Cancer; ORDET, Ormoni e Dieta nella Eziologia dei Tumori; SMC, Swedish Mammography Cohort

^1^The most important factors included age, BMI, energy intake. If a study adjusted for any of these three factors, it acquired one score

^2^Any additional factor is defined as any factor presented in Table 1 (confounding factors adjusted for in the multivariable analysis), but not including the above three most important factors. If a study adjusted for any of these additional factors, it acquired one score

### Associations between dietary patterns and the risk of breast cancer

**Fig. 2:**
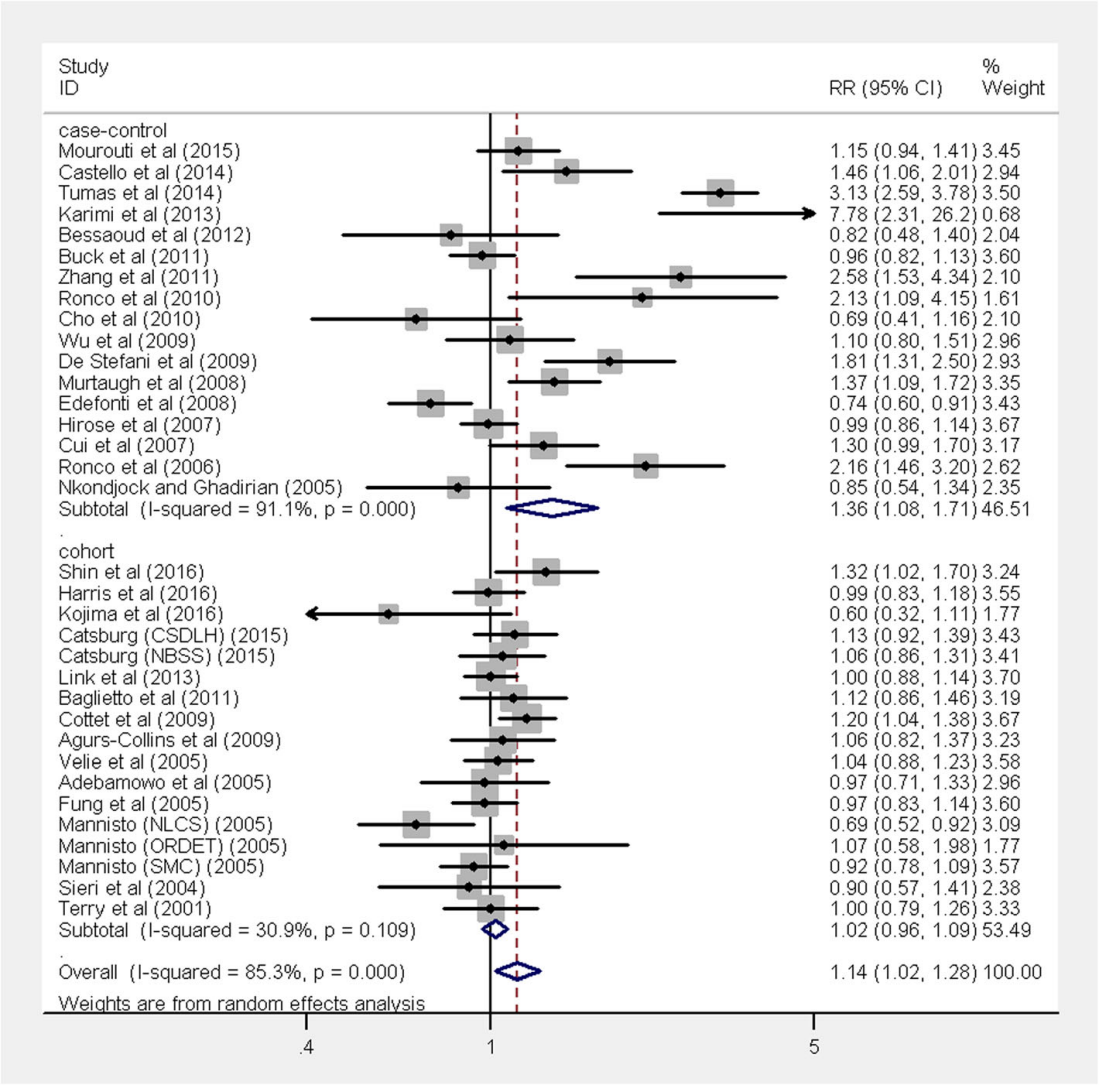
Forest plot shows the association between the highest category of a Western dietary pattern and the risk of breast cancer

The multivariable-adjusted RRs for each study and the combined RR for the highest compared with the lowest categories of a Western dietary pattern are shown in Fig. 2. The pooled result found a positive association between the Western dietary pattern and the risk of breast cancer (RR 1.14, 95% CI 1.02, 1.28, *P* = 0.017), with significant heterogeneity (*I*^2^ = 85.3%, *P* < 0.001). The positive association was only significant in case-control studies (RR 1.36, 95% CI 1.08, 1.71, *P* < 0.001) with significant heterogeneity (*I*^2^ = 91.1%, *P* < 0.001), but not in cohort studies (RR 1.02, 95% CI 0.96, 1.09, *P* = 0.49) with no evidence of significant heterogeneity (*I*^2^ = 30.9%, *P* = 0.109).

**Fig. 3:**
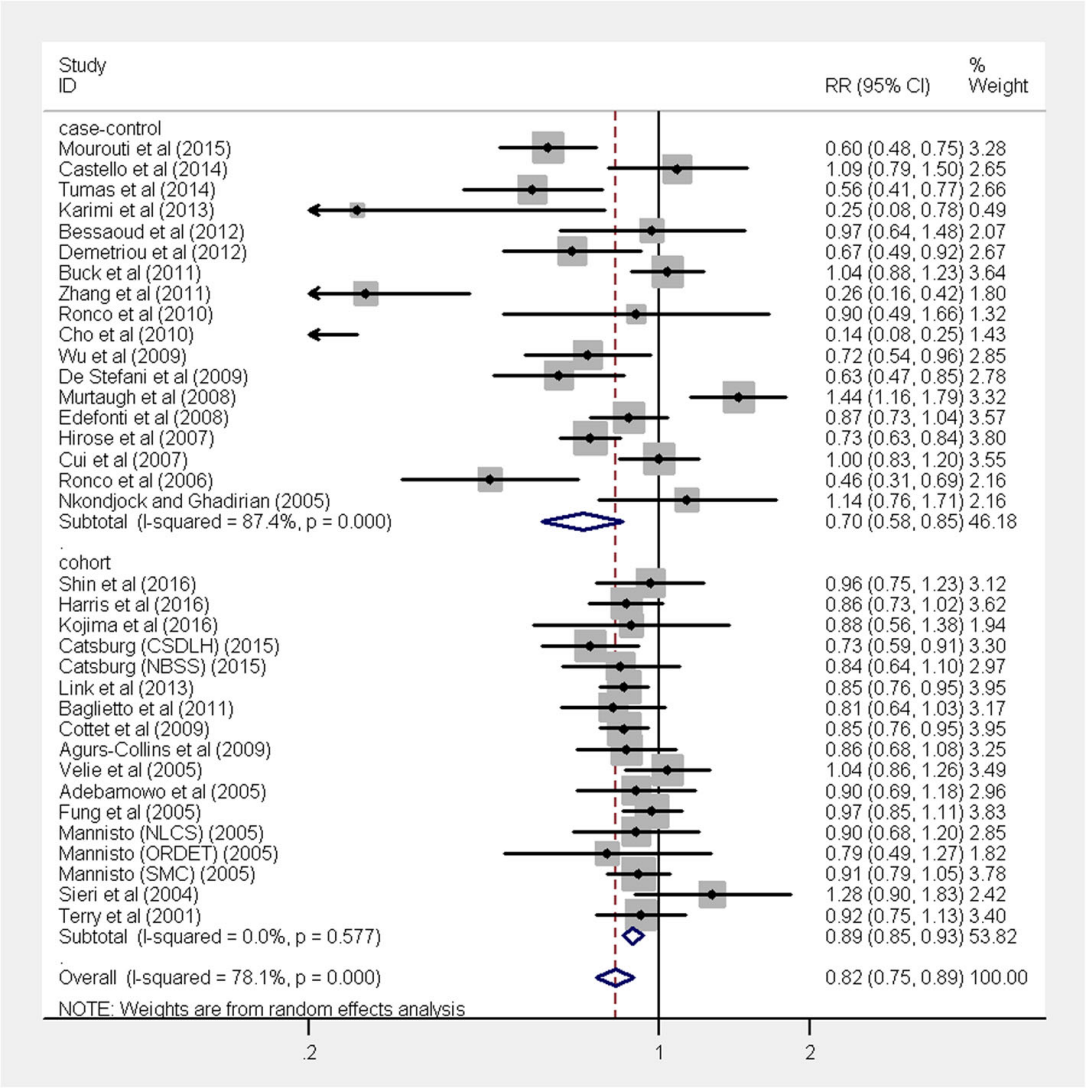
Forest plot shows the association between the highest category of a prudent dietary pattern and the risk of breast cancer

Overall, the combined RR of breast cancer for the highest compared with the lowest category of a prudent pattern was 0.82 (95% CI 0.75, 0.89; *P* < 0.001), with large heterogeneity (*I*^2^ = 78.1%, *P* < 0.001) (Fig. 3). A significantly reduced risk of breast cancer was observed in case-control studies (RR 0.70, 95% CI 0.58, 0.85, *P* < 0.001), with large heterogeneity (*I*^2^ = 87.4%, *P* < 0.001). In addition, an inverse association between prudent dietary and breast cancer risk was also observed in cohort studies (RR 0.89, 95% CI 0.85, 0.93, *P* < 0.001), with no heterogeneity (*I*^2^ = 0.0%, *P* = 0.58).

### Dietary patterns and the risk of breast cancer by menopausal status

**Fig. 4:**
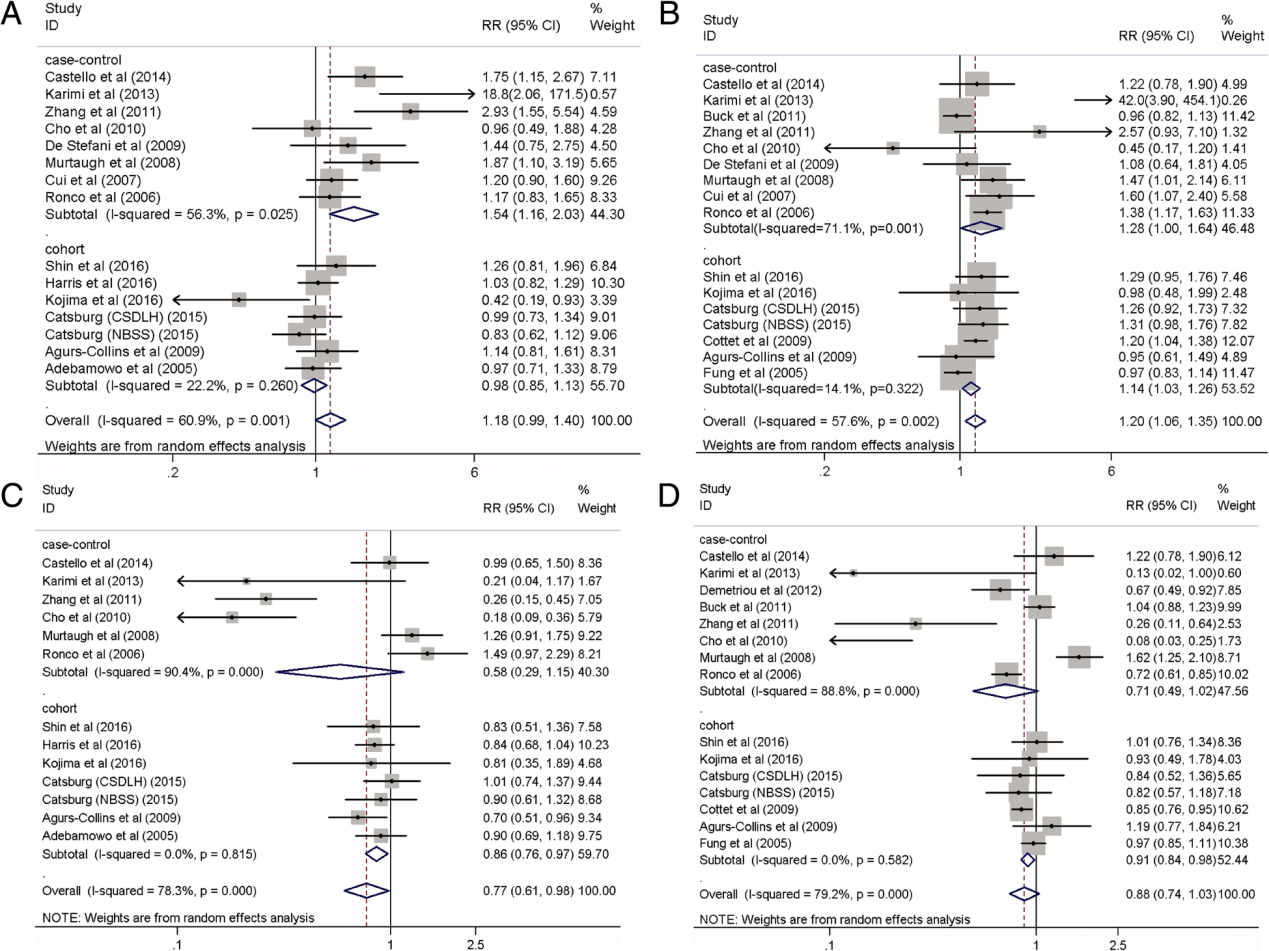
Forest plot shows the association between dietary patterns and the risk of breast cancer by menopause status. **a** Western dietary pattern in premenopausal women. **b** Western dietary pattern in postmenopausal women. **c** Prudent dietary pattern in premenopausal women **d** Prudent dietary pattern in postmenopausal women

Because breast cancers diagnosed at premenopausal stage are different from those diagnosed at postmenopausal stage, stratified analysis was performed to examine whether the association between dietary patterns and breast cancer risk differs by menopausal status. No significant association between a Western dietary pattern and breast cancer risk was observed among premenopausal women (15 studies, RR 1.18, 95% CI 0.99, 1.40, *P* = 0.058) **(**Fig. 4A), with significant heterogeneity (*I*^2^ = 60.9%, *P* = 0.001). However, a significantly increased risk of breast cancer was found among postmenopausal women (16 studies, RR 1.20, 95% CI 1.06, 1.35, *P* = 0.004) (Fig. 4B), with significant heterogeneity (*I*^2^ = 57.6%, *P* = 0.002). In contrast, a significant inverse association comparing the highest to the lowest category of prudent dietary patterns and breast cancer risk was observed among premenopausal women (13 studies, RR 0.77, 95% CI 0.61, 0.98, *P* = 0.034; *I*^2^ = 78.3%, *P* < 0.001; Fig. 4C), but not among postmenopausal women (15 studies, RR 0.88, 95% CI 0.74, 1.03, *P* = 0.112; *I*^2^ = 79.2%, *P* < 0.001; Fig. 4D).

### Dietary patterns and the risk of breast cancer by hormone receptor status

**Fig. 5:**
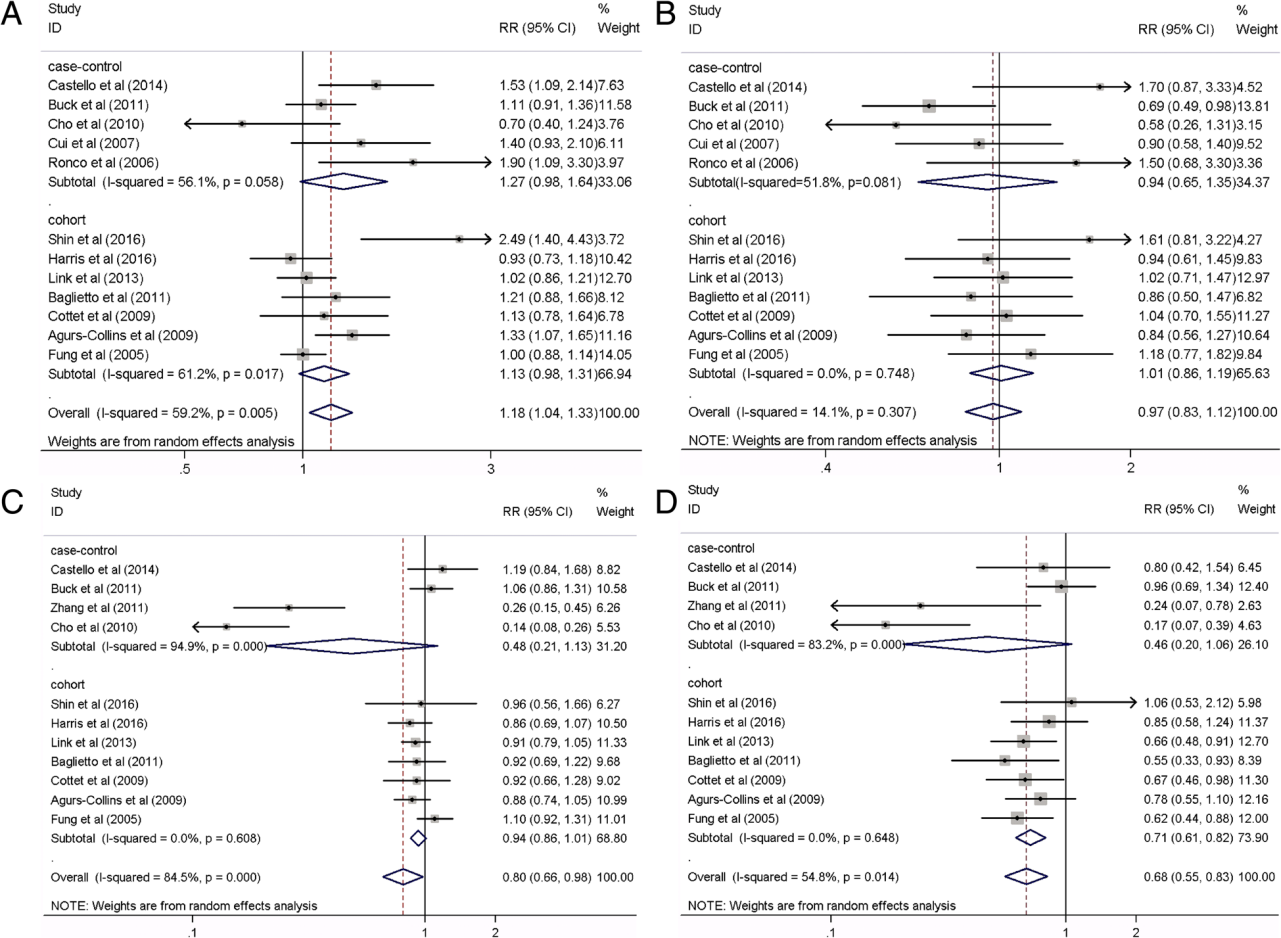
Forest plot shows the association between dietary patterns and the risk of breast cancer by hormone receptor status. **a** Western dietary pattern in breast cancer patients with estrogen and/or progesterone receptor positive. **b** Western dietary pattern in breast cancer patients with estrogen and/or progesterone receptor negative. **c** Prudent dietary pattern in breast cancer patients with estrogen and/or progesterone receptor positive. **d** Prudent dietary pattern in breast cancer patients with estrogen and/or progesterone receptor negative

The Western dietary pattern was significantly associated with an 18% increase in the risk of estrogen receptor (ER+) and/or progesterone (PR+) breast tumors (12 studies, RR 1.18, 95% CI 1.04, 1.33, *P* = 0.012; *I*^2^ = 59.2%, *P =* 0.005; Fig. 5A). However, no association was found for the ER− and/or PR− tumors (12 studies, RR 0.97, 95% CI 0.83, 1.12, *P* = 0.671; *I*^2^ = 14.1%, *P* = 0.307; Fig. 5B). In addition, the prudent dietary pattern was significantly associated with a 20% reduction in the risk of ER+ and/or PR+ tumors (11 studies, RR 0.80, 95% CI 0.66, 0.98, *P* = 0.03; *I*^2^ = 84.5%, *P* < 0.001; Fig. 5C) and 32% reduction in the risk of ER− and/or PR− tumors (11 studies, RR 0.68, 95% CI 0.55, 0.83, *P* < 0.001; *I*^2^ = 54.8%, *P =* 0.014; Fig. 5D), respectively. Further meta-analyses according to ER subtypes within strata of menopausal status found no significant association between Western or prudent dietary patterns and breast cancer risk (Table 3).

**Table 3:** Meta-analyses on the association between dietary patterns and breast cancer risk according to ER subtypes within strata of menopausal status

	Premenopausal						Postmenopausal					
	No. of studies	RR (95% CI)	*P*	Heterogeneity *χ*^2^	*I*^2^ (%)	*P*_Heterogeneity_	No. of studies	RR (95% CI)	*P*	Heterogeneity *χ*^2^	*I*^2^ (%)	*P*_Heterogeneity_
Prudent												
ER+ and/or PR +	2	0.39(0.08, 1.88)	0.243	14.15	92.9	< 0.001	6	0.87(0.69, 1.09)	0.246	22.06	77.3	0.001
ER− and/or PR−	2	0.47(0.13, 1.70)	0.255	3.87	74.1	0.049	5	0.72(0.49, 1.06)	0.102	13.15	69.6	0.011
Western												
ER+ and/or PR+	3	1.08(0.83, 1.41)	0.532	2.36	15.3	0.307	7	1.15(0.99, 1.34)	0.057	10.26	41.5	0.114
ER− and/or PR−	3	0.94(0.67, 1.33)	0.760	0.13	0	0.936	6	0.91(0.81, 1.01)	0.322	7.29	31.4	0.200

ER, estrogen receptor; PR, progesterone

### Subgroups, meta-regression, and sensitivity analyses

Table 4 presents the results of the subgroup analyses. In stratified analyses by study design, number of cases, geographical location, number of adjusted covariates, study quality score, and with or without adjustment for several confounders, we found that the Western dietary pattern was positively associated with the risk of breast cancer in some subgroups with significant heterogeneity in the subgroups. In comparison, the prudent dietary pattern was inversely associated with the risk of breast cancer among the subgroups also with significant heterogeneity. However, no evidence of heterogeneity was observed among the subgroups with meta-regression analyses.

**Table 4:** Meta-analyses on the association between dietary patterns and breast cancer risk

Subgroups	Western							Prudent						
	No. of studies	RR (95% CI)	*P*	Heterogeneity *χ*^2^	*I*^2^ (%)	*P*_Heterogeneity_	*P*_interaction_	No. of studies	RR (95% CI)	*P*	Heterogeneity *χ*^2^	*I*^2^ (%)	*P*_Heterogeneity_	*P*_interaction_
Overall	34	1.14(1.02, 1.28)	0.017	225.1	85.3	< 0.001		35	0.82(0.75, 0.89)	< 0.001	155.1	78.1	< 0.001	
Study design														
Case-control	17	1.35(1.08, 1.71)	0.009	180.3	91.1	< 0.001	0.021	18	0.70(0.59, 0.85)	< 0.001	135.3	87.4	< 0.001	0.091
Cohort	17	1.02(0.95, 1.09)	0.490	23.2	30.9	0.109		17	0.89(0.85, 0.93)	< 0.001	14.3	0.0	0.577	
No of cases														
< 1000	16	1.34(1.03, 1.76)	0.031	128.8	88.4	< 0.001	0.050	17	0.66(0.54,0.82)	< 0.001	88.8	82.0	< 0.001	0.023
> 1000	18	1.03(0.96, 1.10)	0.43	40.1	57.6	0.001		18	0.90(0.84, 0.97)	0.006	44.0	61.4	< 0.001	
Geographical location														
Europe	11	0.97(0.86, 1.11)	0.723	30.2	66.9	0.001	< 0.001	12	0.88(0.80, 0.97)	0.014	25.1	56.2	0.009	< 0.001
North America	11	1.04(0.98, 1.11)	0.194	8.73	0	0.558		11	0.91(0.82, 1.02)	0.123	29.8	66.5	0.001	
South America	4	2.33(1.70, 3.19)	< 0.001	9.74	69.2	0.021		4	0.58(0.47, 0.72)	< 0.001	3.56	15.7	0.313	
Asia	8	1.21(0.93, 1.56)	0.143	32.6	78.6	< 0.001		8	0.58(0.42, 0.80)	0.001	68.6	89.9	< 0.001	
No of adjusted covariates														
≤ 10	16	1.27(1.01, 1.63)	0.048	173.5	91.4	< 0.001	0.226	16	0.74(0.64, 0.86)	< 0.001	63.5	76.4	< 0.001	0.279
> 10	18	1.06(0.98, 1.15)	0.127	40.6	58.1	< 0.001		19	0.87(0.78, 0.96)	0.009	83.7	78.5	< 0.001	
Study quality score														
≤ 7	18	1.25(1.04, 1.48)	0.017	160.4	89.4	< 0.001	0.190	19	0.77(0.67, 0.89)	0.001	122.2	85.3	< 0.001	0.541
> 7	16	1.02(0.92, 1.14)	0.644	42.9	65.1	< 0.001		16	0.85(0.78, 0.93)	0.001	32.7	54.2	0.005	
Adjustment for confounders														
Age														
Yes	24	1.07(0.98, 1.18)	0.152	84.4	72.8	< 0.001	0.233	25	0.85(0.77, 0.93)	0.001	105.9	77.3	< 0.001	0.502
No	10	1.29(0.96, 1.74)	0.094	105.8	91.5	< 0.001		10	0.74(0.62, 0.91)	0.004	39.4	77.2	< 0.001	
BMI														
Yes	31	1.12(1.01, 1.26)	0.043	211.6	85.8	< 0.001	0.426	32	0.81(0.75, 0.89)	< 0.001	138.4	77.6	< 0.001	0.754
No	3	1.37(0.88, 2.13)	0.158	8.66	76.9	0.013		3	0.85(0.52, 1.40)	0.526	15.2	86.9	< 0.001	
Energy														
Yes	31	1.16(1.02, 1.31)	0.023	222.4	86.5	< 0.001	0.754	31	0.84(0.77, 0.92)	< 0.001	133.7	77.6	< 0.001	0.340
No	3	1.05(0.94, 1.16)	0.416	1.51	0.0	0.470		4	0.69(0.62, 0.77)	< 0.001	2.24	0.0	0.524	
Smoking														
Yes	22	1.12(1.01, 1.25)	0.040	70.5	70.2	< 0.001	0.844	22	0.82(0.72, 0.93)	0.003	128.4	83.7	< 0.001	0.853
No	12	1.16(0.93, 1.48)	0.193	153.5	92.8	< 0.001		13	0.82(0.75, 0.89)	< 0.001	25.5	52.9	0.013	
Alcohol														
Yes	14	0.99(0.91, 1.08)	0.854	23.5	44.6	0.036	0.075	14	0.84(0.74, 0.95)	0.007	52.9	75.4	< 0.001	0.827
No	20	1.271.07, 1.50)	0.005	179.4	89.4	< 0.001		21	0.80(0.72, 0.90)	< 0.001	101.9	80.4	< 0.001	
Family history of breast cancer														
Yes	28	1.06(0.98, 1.16)	0.140	88.5	69.5	< 0.001	0.069	29	0.82(0.75, 0.90)	< 0.001	136.5	79.5	< 0.001	0.942
No	6	1.44(0.94, 2.20)	0.090	68.6	92.7	< 0.001		6	0.79(0.64, 0.98)	0.032	16.5	69.7	0.006	
Physical activity														
Yes	24	1.19(1.04, 1.36)	0.012	167.5	86.3	< 0.001	0.409	24	0.82(0.74, 0.91)	< 0.001	118.4	80.6	< 0.001	0.985
No	10	1.05(0.87, 1.26)	0.628	45.4	80.2	< 0.001		11	0.81(0.71, 0.94)	0.004	36.5	72.6	< 0.001	
Hormone use														
Yes	13	1.04(0.94, 1.14)	0.457	27.9	57.0	0.006	0.121	14	0.85(0.75, 0.97)	0.016	67.8	80.8	< 0.001	0.649
No	21	1.26(1.05, 1.51)	0.011	191.3	89.5	< 0.001		21	0.79(0.71, 0.89)	< 0.001	83.1	75.9	< 0.001	

BMI, body mass index

Sensitivity analyses were also conducted to determine whether the results would change when one study was removed at a time. The results were fairly robust. The summary estimates ranged from 1.08 (95% CIs 1.01, 1.18) to 1.16 (95% CIs 1.04, 1.30) for the Western dietary pattern (Additional file 2: Figure S1) and from 0.81 (95% CIs 0.74, 0.88) to 0.84 (95% CIs 0.78, 0.91) for the prudent dietary pattern (Additional file 3: Figure S2). When the two studies [10, 11] that used diet history questionnaires were excluded, the Western dietary pattern was still significantly associated with an increased (RR 1.14, 95% CI 1.01, 1.28, *P* = 0.036) and the prudent dietary pattern was significantly associated with a reduced risk of breast cancer (RR 0.80, 95% CI 0.73, 0.87, *P* < 0.001), respectively. In addition, when the five studies [13, 14, 59, 64, 72] with the low quality score of less than seven were removed, the results did not materially change.

### Publication bias

Although the funnel plot was slightly asymmetric, after using the trim-and-fill method, visual inspection of Begg’s funnel plot did not identify substantial asymmetry (Additional file 4: Figure S3 and Additional file 5: Figure S4). In addition, Begg’s and Egger’s tests showed no evidence of publication bias for Western dietary pattern studies (Begg’s test *P* = 0.138, Egger’s test *P* = 0.347). Although there was marginally significant publication bias for prudent dietary pattern studies (Begg’s test *P* = 0.088, Egger’s test *P* = 0.049), no evidence of publication bias was observed after using the trim-and-fill method (Begg’s test *P* = 0.687, Egger’s test *P* = 0.975), and the association remained significant (RR 0.87, 95% CI 0.79, 0.97, *P* = 0.009).

## Discussion

This meta-analysis of 32 observational studies including 43,285 breast cancer cases supports a positive association of a Western dietary pattern and an inverse association of a prudent dietary pattern with the risk of breast cancer. The Western dietary pattern was overall associated with a 14% increased risk, when comparing high vs. low groups, which was only significant in case-control studies (35% increased risk) but not in cohort studies (2% increase), suggesting that recall bias might at least partially explain the discrepant results in different study designs. The prudent dietary pattern, comparing high vs. low groups, was associated with a reduced risk (overall 18% decrease) of breast cancer in both case-control (30% decreased risk) and cohort studies (11% decrease). Furthermore, results of a Western dietary pattern are null in study designs with greater covariate adjustment and higher study quality, suggesting that the observed association may be limited to study designs with more limitations. Other stratified-analyses showed that the positive association between a Western dietary pattern and breast cancer risk was statistically significant among postmenopausal women, but not significant among premenopausal women. In contrast, the inverse association between a prudent dietary pattern and the risk of breast cancer was significant among premenopausal women, but not significant among postmenopausal women. In addition, the Western dietary pattern was significantly associated with an increased risk of ER+ and/or PR+, but not ER− and/or PR− breast tumors. In comparison, the prudent dietary pattern was significantly associated with a lowered risk of both ER+ and/or PR+ and ER− and/or PR− tumors.

In 2007, the WCRF report concluded that there was insufficient evidence to make a judgment about the relationship between dietary patterns and the risk of breast cancer [9]. Subsequently, a meta-analysis published in 2010 including eight case-control and eight cohort studies showed that a prudent/healthy dietary pattern was associated with an 11% reduction in the risk of breast cancer, whereas no association was found between a Western/unhealthy dietary pattern and breast cancer risk [27]. The results from our analyses are consistent with those of the previous meta-analysis to some extent. The 18% reduction in breast cancer risk associated with the prudent dietary pattern was stronger than the result in the previous meta-analysis. We also found a 14% increase in breast cancer risk associated with the Western dietary pattern. The result of a positive association between the Western dietary pattern and breast cancer risk in case–control but not cohort studies is also in line with the finding of subgroup analyses in the previous meta-analysis. However, an inverse association between the prudent dietary pattern and the risk of breast cancer was observed only in cohort but not case-control studies in that meta-analysis, whereas an inverse association was observed in both case-control and cohort studies in our meta-analysis. With an additional 10 case-control [15, 62–70] and 6 cohort studies [13, 17, 52, 77–79] and more than double the number of breast cancer cases (*n* = 43,285 vs. 19,908 cases) than in the previous meta-analysis, the current study had greater statistical power to detect significant associations.

Because estrogens have long been hypothesized to play an essential role in breast cancer development and the source and metabolic pathway of estrogens are different between premenopausal and postmenopausal women [80], the etiology and risk factors of breast cancer differs by menopausal status. Hence, we further conducted stratified analysis to examine the dietary pattern-breast cancer association by menopausal status. Interestingly, the Western dietary pattern was associated with a 20% increased risk of breast cancer among postmenopausal but not among premenopausal women. In contrast, the prudent dietary pattern was associated with a 23% reduction in breast cancer risk among premenopausal but not among postmenopausal women. The different associations between dietary patterns and breast cancer risk by menopausal status may be explained by the diet-estrogen pathway. After menopause, when ovarian production of estrogen ceases, the serum levels of estrogen come from aromatization of androstenedione to estrone in the stroma of fat cells followed by conversion to estradiol; therefore, adipose tissue is the major source of estrogen among postmenopausal women [81]. Obese postmenopausal women have both relatively high serum concentrations of estradiol and an increased risk of breast cancer [82]. Our results also showed that adjustment for BMI attenuated the magnitude of the positive Western dietary pattern-breast cancer risk. Therefore, one plausible mechanism that the Western dietary pattern, characterized by high intakes of energy, red meat and processed meat, and animal fat, can increase breast cancer risk is through increased BMI and increased levels of estrogen, and particularly among postmenopausal women. These explanations need to be further examined in future studies. As diet is advocated by the WCRF as a potentially modifiable means to reduce cancer risk, the prudent dietary pattern should be adopted, particularly among younger premenopausal women, to protect against the development of breast cancer. The prudent dietary pattern is characterized by high intakes of fruit, vegetables, and whole grains. Fruits and vegetables contain a variety of micronutrients with anti-cancer properties, including antioxidant vitamins such as vitamin E and vitamin C, folate, dietary fiber, dithiolthiones, isothiocyanates, glucosinolates, indoles, protease inhibitors, and phytochemicals (lycopene, phenolic compounds, and flavonoids). These nutrients may influence carcinogenic process by affecting the immune system and oxidative stress, altering hormonal status, modifying the structure and function of cell membranes, and modulating cell signaling transduction pathways and gene expression [83, 84]. The inverse association between a prudent dietary pattern and breast cancer observed among premenopausal women may be due to the high estrogen levels and potentially stronger protective effect of various nutrients rich in fruits and vegetables of this dietary pattern.

Hormone receptor status is an important diagnostic and prognostic characteristic of breast tumor and, therefore, merits consideration. Among the studies which examined the dietary patterns and breast cancer association by hormone receptor status [10, 13, 16, 17, 58, 62, 64, 67, 74, 76–78], the Western dietary pattern has been found to be associated with an increased risk of hormone receptor-positive breast tumors in some studies [58, 67, 76, 78], whereas no association was found regardless of hormone receptor status in some other studies [13, 17, 74]. Conflicting results were also reported for the association between the prudent dietary pattern and breast cancer risk by hormone receptor status. In our stratified analyses, the Western dietary pattern was associated with an 18% increased risk of ER- and/or PR-positive tumors but not ER- and/or PR-negative tumors. In contrast, the prudent dietary pattern was associated with a 20% reduced risk of ER- and/or PR-positive tumors and 32% reduced risk of ER- and/or PR-negative tumors, respectively. The prudent dietary pattern may play an important role in estrogen metabolism and breast cancer protection, as it is characterized by high intakes of fruit, vegetables, and whole grains, which are rich sources of phytoestrogens, isothiocyanates, flavonoids, antioxidants, and folate, all of which have been found to be associated with a reduced breast cancer risk [85, 86]. The positive association between the Western dietary pattern and hormone receptor-positive tumors is consistent with the results from a previous intervention study which found that decreased fat intake was associated with risk reduction mainly in ER+ tumors [87].

Our study had several strengths. With a larger number of studies and breast cancer cases than the previous meta-analysis, our meta-analysis had more statistical power to detect a significant association between the Western dietary pattern and breast cancer risk and to calculate a more reliable estimate for the prudent dietary pattern and breast cancer association. In addition, to the best of our knowledge, this is the first meta-analysis to analyze the dietary pattern and breast cancer risk by menopausal status and hormone receptor status. We also carried out sensitivity analyses to show that the results were fairly robust.

Potential limitations of this study should also be considered. First, the quality of meta-analyses is largely dependent on the quality of the original studies included in the meta-analyses. The current meta-analysis included 18 case-control studies and 14 cohort studies. Therefore, the possibility of recall bias related to differential recalls of dietary intake between cases and controls and control selection bias in case-control studies cannot be completely ruled out. Due to a lack of significance in association between a Western dietary pattern and breast cancer in cohort studies, the significant association in case-control studies may not be a true association as a result of recall bias. Second, unmeasured and uncontrolled confounding is always of a concern in observational studies, although most included studies adjusted a large number of factors which may potentially confound the dietary patterns and breast cancer association. However, not all potential confounders were adjusted for in every study, such as breast density [88] and history of chest exposure to high doses of radiation [89]. Third, because the two dietary patterns were identified and classified differently in the studies reviewed, it is also possible that the two dietary patterns may be misclassified and the results may be influenced. To minimize potential misclassification, we selected only the most commonly identified dietary patterns across studies and ensured as far as possible that the dietary patterns were similar with regard to factor loadings of foods most commonly consumed. Furthermore, we estimated the summary RRs comparing the highest category of the particular dietary patterns to the lowest category. Fourth, the FFQs and diet histories were used to assess dietary patterns in the studies. Although the reproducibility and validity of these methods was reported [90], the variability in the factor analysis and/or principal component analysis may still exist [91]. As a result of the uncorrelated data-driven patterns, only certain aspects of diet were captured within a given pattern. For example, a prudent pattern often had factor loading near 0 for animal products; thus, these diet high in both plants and animal products were not penalized for consuming some processed/animal products that may be harmful. Finally, publication bias was found among the studies which reported a prudent dietary pattern. However, after using a trim-and-fill method, the inverse association remained significant.

## Conclusions

In summary, our meta-analysis provides potential evidence of a possible positive association between the Western dietary pattern and an inverse association between the prudent dietary pattern and breast cancer risk. However, the results should be interpreted with caution, as the observed positive association may be limited to study designs with more limitations. Subgroup analyses found that these associations differed by menopausal status and hormone receptor status. As diet is potentially modifiable, the findings may have important implications to promote a prudent dietary pattern for breast cancer prevention.

## Additional files

Additional file 1: PRISMA checklist. (DOC 71 kb)

Additional file 2: **Figure S1.** Sensitivity analysis of the associations between a Western dietary pattern and the risk of breast cancer by eliminating one study at a time. (JPG 190 kb)

Additional file 3: **Figure S2.** Sensitivity analysis of the associations between a prudent dietary pattern and the risk of breast cancer by eliminating one study at a time. (JPG 190 kb)

Additional file 4: **Figure S3.** Funnel plots of associations between a Western dietary pattern and risk of breast cancer. (JPG 34 kb)

Additional file 5: **Figure S4.** Funnel plots of associations between a prudent dietary pattern and risk of breast cancer. On the left is the plot before using the trim-and-fill method, and on the right is the plot after using the trim-and-fill method. The boxes represent the filled studies. (JPG 82 kb)

## Abbreviations

BMI: Body mass index
CIs: Confidence intervals
ER: Estrogen receptor
FFQ: Food-frequency questionnaire
HRs: Hazards ratios
IRRs: Incidence rate ratios
NOS: Newcastle Ottawa Scale
ORs: Odds ratios
PR: Progesterone
RRs: Relative risks
