# Supplementary figures and images for: Associations between dietary patterns and the risk of breast cancer: a systematic review and meta-analysis of observational studies
Source: Breast Cancer Res. 2019 Jan 29;21:16. doi: 10.1186/s13058-019-1096-1 (PMC6352362; doi:10.1186/s13058-019-1096-1)

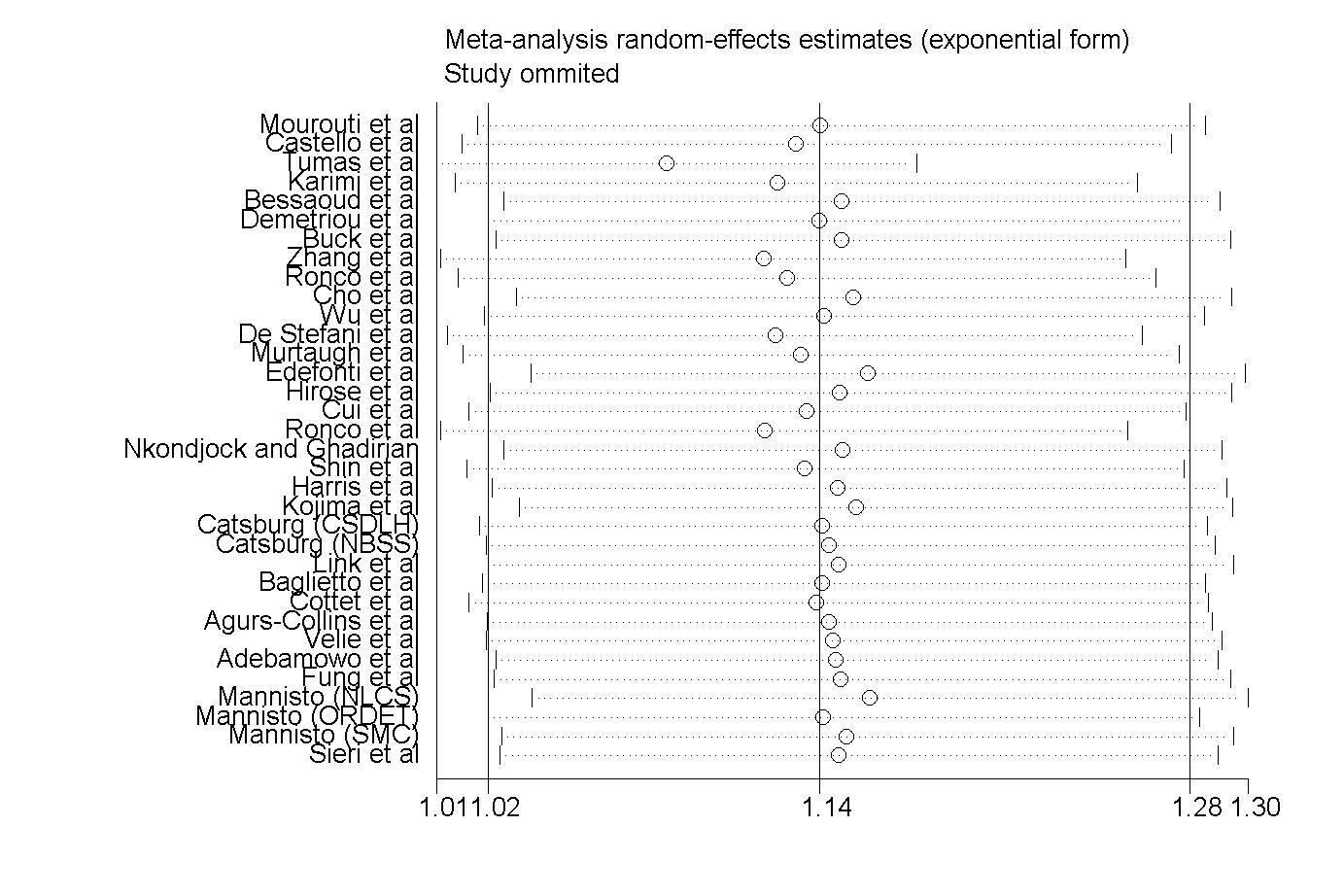

Supplement: Supplementary file 2 — Figure S1. Sensitivity analysis of the associations between a Western dietary pattern and the risk of breast cancer by eliminating one study at a time. (JPG 190 kb) [file 13058_2019_1096_MOESM2_ESM.jpg]

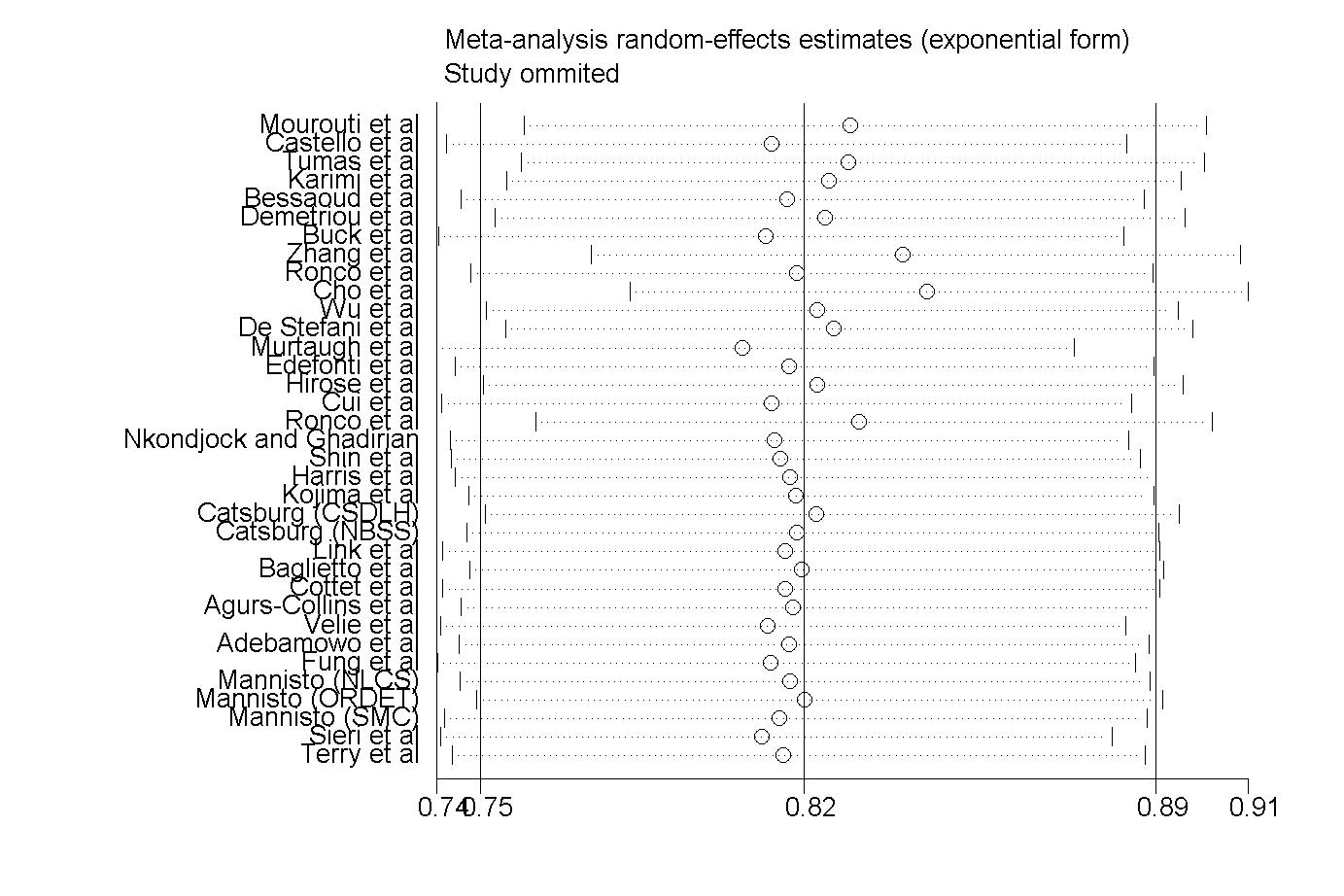

Supplement: Supplementary file 3 — Figure S2. Sensitivity analysis of the associations between a prudent dietary pattern and the risk of breast cancer by eliminating one study at a time. (JPG 190 kb) [file 13058_2019_1096_MOESM3_ESM.jpg]

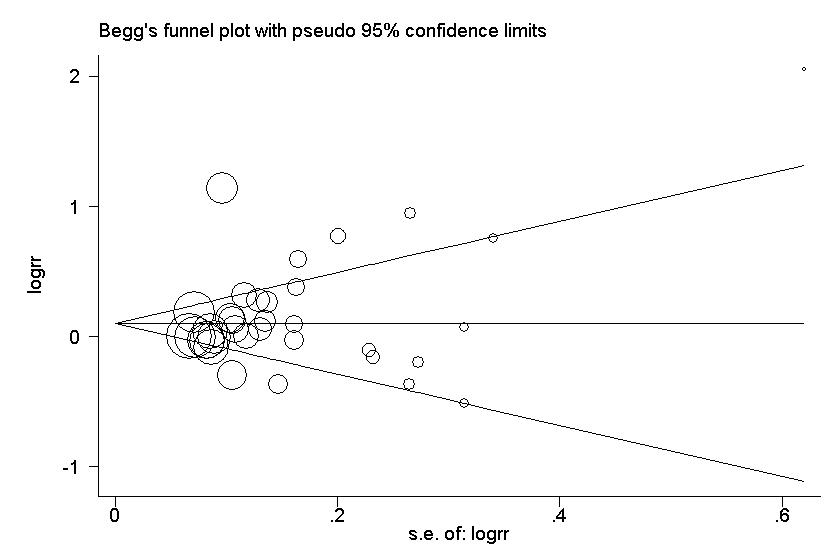

Supplement: Supplementary file 4 — Figure S3. Funnel plots of associations between a Western dietary pattern and risk of breast cancer. (JPG 34 kb) [file 13058_2019_1096_MOESM4_ESM.jpg]

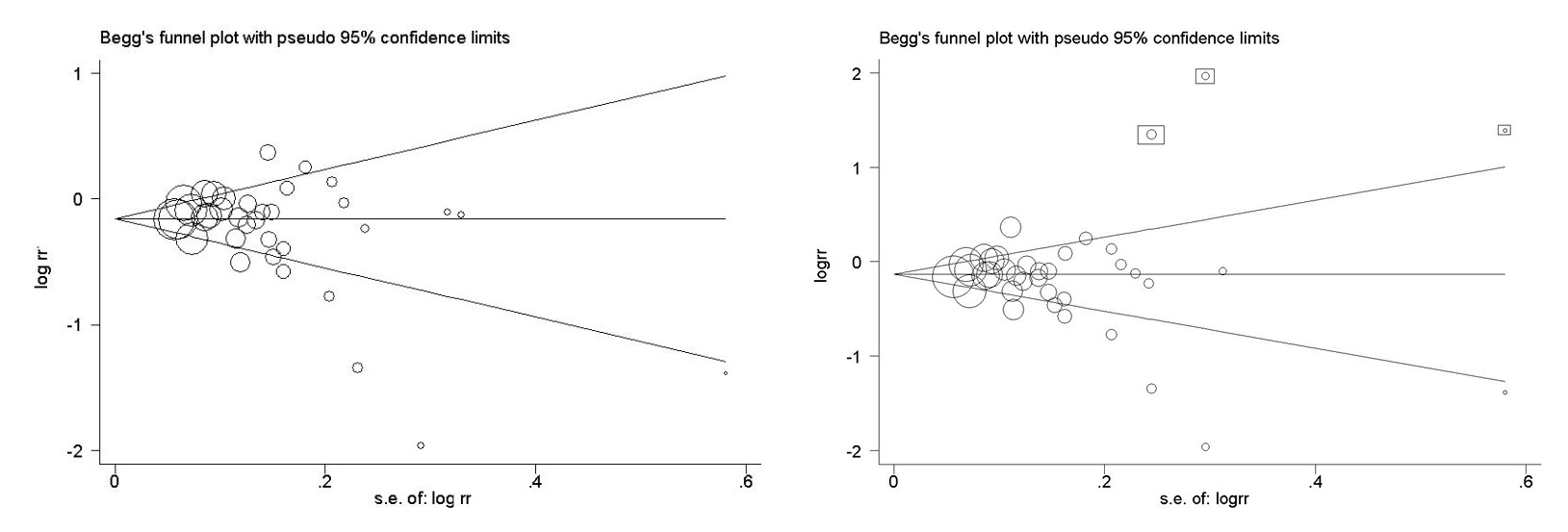

Supplement: Supplementary file 5 — Figure S4. Funnel plots of associations between a prudent dietary pattern and risk of breast cancer. On the left is the plot before using the trim-and-fill method, and on the right is the plot after using the trim-and-fill method. The boxes represent the filled studies. (JPG 82 kb) [file 13058_2019_1096_MOESM5_ESM.jpg]
